# More *versus* less: the unresolved debate on the best surgical approach to temporal lobe epilepsy with hippocampal sclerosis

**DOI:** 10.1055/s-0043-1771265

**Published:** 2023-07-26

**Authors:** André Palmini

**Affiliations:** 1 Pontificia Universidade Católica do Rio Grande do Sul, Escola de Medicina, Núcleo de Neurociências Clínicas, Porto Alegre RS, Brazil.; 2 Pontificia Universidade Católica do Rio Grande do Sul, Hospital São Lucas, Serviço de Neurologia & Programa de Cirurgia da Epilepsia, Porto Alegre RS, Brazil.

People with epilepsy suffer from the unexpectedness of seizures, leading to a constant state of tension, but also from the psychosocial obstacles related to recurrent attacks in all kinds of social and professional environments. The ideal scenario in which seizures are completely controlled with antiseizure medications is not a reality for about 40% of patients, and the picture is even bleaker for those with focal onset seizures from specific structural etiologies. 1 Facing such patients, neurologists must keep in mind the possibility that resective surgery may be a game changer, controlling seizures and opening avenues of psychosocial opportunities for people usually kept at the fringes of society for a long time. 2

Perhaps there is no better example of a medically refractory, yet surgically remediable, epilepsy syndrome than temporal lobe epilepsy with hippocampal sclerosis (TLE/HS). 3 4 People with this type of epilepsy are intellectually normal and usually begin with recurrent seizures in adolescence or early adulthood. They often refer a visceral-autonomic, emotional, or mnemonic warning (aura), followed by disconnection from the environment and automatic behavior that extends for a minute or so – long enough to incur in car accidents, burning or other injuries and to compromise social and professional encounters. Thus, although generalized motor attacks are uncommon, seizures carry significant risk. MRI points to the atrophic, sclerotic hippocampus, interictal scalp EEGs show antero-basal and middle temporal epileptiform discharges, often with unilateral predominance, and seizures on scalp are recorded from the temporal lobe harboring the sclerotic hippocampus. 5 6

The epileptogenic lesion is centered in the atrophic hippocampus and the circuitry involved in the seizures is now very well defined, with the implication of the adjacent parahippocampal gyrus and the amygdala in the epileptogenic zone now established. Unquestionably, these mesial temporal structures must be resected to achieve seizure control. *However, whether lateral and anterior temporal (polar) cortical regions are part of the epileptogenic circuit in TLE/HS and should be included in the resection is still debatable* . 7

At first glance, for the non-specialist, this might seem an odd debate. That person would think that one would only proceed with surgery for seizures with full knowledge of the structures involved in seizure generation and that presurgical evaluation procedures should provide the necessary reassurance. That, of course, would need a method to study such structures directly with intracerebral electrodes. Such method – stereo-electroencephalography (SEEG) with stereotactically-implanted electrodes – does exist, but at high cost and some risk. Furthermore, because the clinical-EEG-MRI picture is so homogeneous in most patients with TLE/HS, and the results are related to complete resection of the mesial structures, the vast majority of patients with this very common form of refractory epilepsy proceed to surgery following non-invasive evaluation.

Importantly, the relevance of the debate about which structures must be resected in this entity is highlighted both by the perspective of very good surgical results when the right thing is done, 8 9 and by the fact that anterior temporal lobe circuitry (mesial and neocortical structures) is implicated in cognitive function (notably memory, but also language in the dominant hemisphere) that could be somewhat compromised by surgery. 10 Up to 70–80% of patients achieve long-term complete seizure control with surgery 8 9 but some patients in these studies had some degree of alteration in memory and naming functions. 10

In direct words, resecting *less* may deprive patients from full seizure control whereas resecting *more* may compromise cognition more than should have been needed to achieve seizure control. Despite many decades of clinical practice and research, the jury is still out: Some studies show a significant difference in seizure control favoring anterior temporal lobectomy (ATL; ie, a more extensive resection, including both the mesial and the neocortical/temporal pole structures), 7 11 whereas others show similar results regarding seizures yet better cognitive outcome with a selective approach, resecting only the mesial structures (selective amygdalo-hippocampectomy - SAH). 3 8 12 13 14

In this issue, Almeida and colleagues present their results in 132 patients with TLE/HS followed for a mean of approximately 5 years. 15 They compare the results in seizure control in the 70 that had an ATL in the right side with the 62 who had a selective procedure (SAH) in the left. The rationale for their approach is clever: because verbal memory plays a bigger role in mnemonic functions, they assumed that a selective approach in the dominant temporal lobe would reduce cognitive risks, while still supported by a significant body of literature showing similar effectiveness with ATL regarding seizure control. Therefore, *more* structures were resected on the right and *less* on the left. Because they did not provide neuropsychological nor quality of life results, their rationale could not be validated in their series, although it makes sense from what the literature says. In practical terms, results were better with the resection of *more* tissue – ie, ATL was more efficacious controlling seizures than SAH.

These results, however, should be seen with caution for several reasons. The first is that this study adds to the vexing scenario of the paucity of randomized controlled studies in epilepsy surgery. Particularly, there are no randomized trials comparing distinct resective strategies in temporal lobe epilepsy, controlling for history and type of initial precipitating insults, lifetime frequency of generalized tonic-clonic seizures, distribution of interictal spikes, occurrence and timing of seizure propagation outside the temporal lobe in the scalp EEG, as well as for MRI variables, such as neocortical deafferentation from white matter vacuolation in the temporal pole. 16 All these features might suggest a more prominent interaction between neocortical and mesial temporal structures and would (theoretically) be best served by a larger resection. The putative imbalance of these variables in the two groups inevitably introduces a selection bias in the results and bring us back to an individual, tailored, approach.

Three additional aspects raised by the results of Almeida and their colleagues – and that could impact in their findings - merit discussion. One is the issue of the currently denominated type IIIa focal cortical dysplasia (FCD). This is a microscopic finding essentially featuring anterior temporal neocortical dyslamination associated with ipsilateral hippocampal sclerosis. 17 How relevant is FCD IIIa to the epileptogenic circuitry is far from clear. Some studies suggest that up to 25% of patients with unilateral HS have associated neocortical dyslamination (ie, FCD IIIa) 18 and should this finding be epileptogenically relevant, one would expect *all comparative studies* to report better results with ATL, and a much larger difference in the efficacy between the two approaches – which is clearly not the case. 3 4 13 Interestingly, the authors apparently did not find a single case of FCD IIIa in the pathological analysis of their 70 consecutive right temporal neocortical resections. This may relate to the methods used for the histopathologcal analyses of the resected tissue.

Then comes the issue of ‘temporal-plus epilepsies. It is not mentioned in the paper which clinical features were considered ‘typical’ of temporal lobe seizures and which semiological manifestations that can also be seen in patients with TLE/HS yet suggest a larger epileptogenic zone were considered ‘atypical’, thus excluding patients from the cohort. Nasopharyngeal, gustatory, auditory, vestibular and bilateral sensory auras, as well as motor manifestations early in the seizure such as clonic deviation of the mouth, head and eyes version, and contralateral tonic posturing - all suggest additional involvement of insular, perisylvian and posterior temporal regions, despite the presence of hippocampal sclerosis and anterior temporal interictal discharges. 19 Because a larger epileptogenic zone is suspected when some of these features are present, defining what is known as ‘temporal plus epilepsies’, the inclusion of patients with some of these features in the SAH group (the group in which *less* was resected) would automatically reduce the chances of surgical success.

Finally comes the role of the neurosurgeon. Interestingly, epilepsy surgery publications almost never consider the ‘surgeon factor’, ie, the possibility that a given surgeon has greater ability with some approaches and not so much with other approaches. Therefore, the possibility that the results are biased by how complete the pre-defined epileptogenic zone was in fact resected must also be kept in mind. Selective amygdalo-hippocampectomy is usually a technically more demanding technique and Almeida and her colleagues did not provide post-operative MRIs of their patients. Thus, the possibility that the less favorable results seen with the selective approach might be due to less complete resection than was planned should also be considered.

Epilepsy surgery is a very rare commodity in emerging countries and every effort to provide access to this potentially curative treatment should be applauded. The authors should be commended for joining the perspective of sustainable epilepsy surgery and doing the best possible job with non-invasive tools to plan and execute temporal lobe epilepsy surgery. In an era where more and more costly sophistication is being proposed for temporal lobe surgery, showing that good results can be obtained by affordable approaches is a must.
